# From the bench to the bedside: BOLDly going where no one has gone before

**DOI:** 10.4103/2152-7806.66621

**Published:** 2010-07-21

**Authors:** Jason S. Hauptman

**Affiliations:** Intellectual and Developmental Disabilities Center and Department of Neurosurgery, Geffen School of Medicine at UCLA, Los Angeles, CA

## A DIGEST OF ORIGINAL, HIGH-IMPACT NEUROSCIENCE FROM AROUND THE WORLD

In the last 30 days, approximately 1000+ neuroscience-related articles have been published and indexed in Pubmed. Therefore, the question must be asked: how would the neurosurgeon possibly be able to keep up with this massive quantity of literature? Even if one were to read 2 articles a day, it could take almost 3 years just to wade through 1 month‘s worth of material! For this reason, Surgical Neurology International has decided to assist the practicing neurosurgeon by selecting a few notable articles from the highest-impact science journals and presenting them in an easily digestible but sufficiently detailed manner. Welcome to “From the Bench to the Bedside.” We hope that you find this useful, and look forward to any constructive feedback.

## THE BOLD SIGNAL AND FMRI: WHAT DOES IT REALLY MEAN?[1]

Functional magnetic resonance imaging (fMRI)has emerged as a useful tool in both basic and clinical neuroscience. The core concept behind fMRI is to couple structural brain imaging with markers that reflect brain activation during tasks or after stimulus application. This allows the scientist or clinician to visualize brain areas that are physiologically active. In clinical neurosurgery, fMRI can be used for preoperative planning, particularly when resections need to be performed in eloquent areas such as the motor cortex or speech areas. In the case of BOLD (blood oxygen level-dependent) signals, changes in cerebral blood flow and metabolism are reflected by positive (read: “active”) or negative (read: “inhibited”) color signals superimposed on the brain region of interest.

An ongoing controversy within the functional imaging field has been whether BOLD signals truly represent the activity of underlying excitatory neurons or whether they could be due to changes in glial activity, inhibitory and excitatory neurons together, or fiber tracts. The problem is, proving what *exactly* is being activated is difficult, and the notion that the BOLD signal is representative of the activity of excitatory neurons is theoretical.

In this study, Lee and colleagues use an optogenetic approach to determine what exactly drives the BOLD signal. Optogenetics is an exciting new approach towards manipulating the activities of subset of neurons within a given area. This is how it works: First, they created animals whose M1 (motor cortex) principal cortical neurons express channelrhodopsin (ChR2). This is accomplished by using a recombinant adenovirus to deliver the ChR2 gene to principal cortical neurons and encourage them to express it. ChR2 is a transmembrane protein that causes the neurons to depolarize when exposed to pulses of a particular wavelength of light. This is accomplished with high temporal resolution, meaning they can control the activity of the neurons with millisecond accuracy.

Then, after stereotactically placing an MRI-compatible fiber-optic cable adjacent to the viral injection site, the animals were placed in a 7T MRI scanner. Most MRI scanners are 1.5-3.0T; so the 7T is only used in a limited number of places for research and gives highly detailed structural images for functional imaging. The authors then pulsed light through the fiber-optic cable to the cortex, where the cells that expressed ChR2 would fire. They found that positive BOLD signals correlated with the delivery of light pulses, and subsequent activation of the principal excitatory cortical neurons transfected with the ChR2 protein. On the other hand, when they used ChR2 to drive parvalbumin-positive inhibitory interneurons in a different set of animals, the result was a negative BOLD signal. This makes sense, since a negative bold signal would represent inhibition of an area.

Two additional characteristics of the BOLD signal also matched observations scientists have made in other fMRI studies where they scan humans responding to a sensory stimulus and visualize the areas of brain that are activated. First, when they depolarized the cortical neurons and produced a BOLD signal, they found that the kinetics of the BOLD signal matched those previously observed in human studies where subjects respond to a stimulus. This means that by artificially depolarizing the neurons using the ChR2, they were able to replicate the effect of those neurons firing spontaneously in a human after being exposed to a sensory stimulus. Second, they found that after they stopped stimulating the cells, there was a decrease in the BOLD signal that mimicked the “undershoot,” a known phenomenon in humans where the BOLD signal decreases following withdrawal of a sensory stimulus. This is thought to reflect a decrease in cerebral blood flow following stimulus withdrawal.

The final component of this study focused on using this animal model to explore the BOLD signal‘s role in mapping functional circuitry; that is, how the BOLD signal can be interpreted with regard to postsynaptic, downstream activation. In this regard, the optogenetic technique has one primary advantage over conventional electrical stimulation for this purpose — the lack of antidromic stimulation of cells with axons passing through the area being stimulated. Antidromic stimulation refers to the phenomenon where after providing an electrical stimulus to a brain area, some current flows towards the cell body and dendrites instead of towards the synapse. The light pulses stimulate the cells that have ChR2 more physiologically; so current flow in the cells is from cell body to synapse.

To map connectivity, they specifically activated corticothalamic projection neurons and examined the resultant thalamic BOLD signal. They not only found that stimulating M1 corticothalamic neurons results in a strong thalamic BOLD signal, but also the thalamic signal has different kinetic properties compared to the cortical signal. The authors also performed the reverse experiment, transfecting thalamocortical neurons with ChR2 and stimulating the thalamus with light. Again, they discovered BOLD signals in the thalamus as well as the ipsilateral cortex. With the help of this experiment, they show that BOLD signals can reflect one-way activity of projection neurons, allowing a scientist to stimulate the neuron at one end and then map the termination of the projection neuron by seeing the area that displays a BOLD signal; the result — a functional cellular map of the entire brain! Cajal himself would be salivating at the notion.

### Key Points:

1. The BOLD signal that indicates increased blood flow in brain areas, in fact, comes from the activation of excitatory neurons. So the increase in activity in the fMRI is likely related to increases in neuron metabolism, which then lead to increases in blood flow to the area, presumably to bring glucose and other nutrients.
2. Positive cortical BOLD-fMRI signals can be created by excitatory neuronal activation, while negative BOLD-fMRI signals can result from the activity of GABAergic interneurons.
3. BOLD signals in subcortical structures (i.e., thalamus) can result from direct activation of excitatory (i.e., corticothalamic) projections.
4. Optogenetic techniques are important new methods of activating subsets of neurons with high temporal and spatial precision, thus having advantages over conventional methods of stimulation.
5. Optogenetics combined with BOLD-fMRI is a powerful technique for mapping functional brain circuitry.

## TRANSCRANIAL PULSED ULTRASOUND: AN ALTERNATIVE TO DBS AND TMS FOR ACTIVATING NEURONS[2]

Neuromodulation is an emerging field in neurosurgery and the clinical neurosciences, where the focus is to manipulate neuron activity to treat functional neurological disorders. The method of neuromodulation most familiar to the neurosurgeon is DBS (deep brain stimulation), where both low- and high-frequency electrical stimulations of deep brain structures are used to improve symptoms of movement and [more recently] psychiatric disorders. DBS has several drawbacks, including the need to surgically implant an electrode and battery pack, the development of tolerance to stimulation parameters, and side effects from inadvertent stimulation of structures adjacent to the intended target. A recent alternative to DBS is transcranial magnetic stimulation, which is noninvasive but suffers from poor spatial resolution (that is, it is difficult to accurately stimulate specific narrow areas) and may induce epilepsy. Methods of neuromodulation that exist in the laboratory include optogenetic approaches and other genetically engineered proteins, but these require viral vectors for transgene delivery and integration. In this paper, the authors explore a novel option in neuromodulation, viz., transcranial pulsed ultrasound.

In this neurotechnique paper by Tufail *et al*., low-frequency, low-intensity transcranial pulsed ultrasound was investigated in the modulation of *in vivo* mouse brain circuits. First, the authors demonstrated that pulsed ultrasound application to the M1 region results in changed local field potentials (reflecting neurons depolarizing) and cortical spiking within 50 ms of stimulus application. Then, the authors gave tetrodotoxin. It blocks the voltage-gated sodium channels so that a neuron cannot depolarize or transfer sodium into the cell. They found that the toxin blocked cell stimulation. Thus, the pulsed ultrasound caused the neurons to fire action potentials just as they would under physiological circumstances; and an electric current was not necessary as with DBS.

To explore the downstream effects of the M1 (motor cortex) stimulation, the authors found that the ultrasound-mediated M1 stimulation results in EMG (Electomyography)-positive muscle contraction. Even more impressively, they were able to isolate particular muscle groups using small acoustic collimators that allow the beam to be tightly focused on a limited number of neurons. This stimulus-response relationship was very consistent, with a failure rate of less than 5% (though as stimuli were delivered more rapidly, the failure rate increased). The authors also found that the EMG response amplitude inversely correlated with stimulus intensity and frequency — that is, lower frequencies and lower intensities resulted in more significant muscle contraction. Using small, 2-mm, acoustic collimators, the authors showed that c-fos activation (a measure of functional neuronal activation) was restricted to the approximate diameter of collimator. This means that the pulsed ultrasound had extremely high spatial resolution, of the order of approximately 2 mm in lateral dimension. This means that low-intensity, low-frequency pulsed ultrasound results in reliable, robust neural circuit activation with high spatial resolution.

The authors comprehensively assessed the safety of transcranial pulsed ultrasound in their animal model. Using systemic fluorescein administration, they demonstrated that the blood-brain barrier was not disrupted. They found no increases in caspase-3–positive cells, indicating enhanced neuroglial apoptosis (or programmed cell death — capsase-3 is a protein that leads the cell down multiple pathways to its death). This was consistent in both low- and high-intensity pulsed ultrasound. They found no changes in the density of synapses, character of postsynaptic densities, area of presynaptic terminals, density of synaptic vesicles, or ultrastructure of neuropil. They have not experienced any adverse neurologic sequelae in any of their nearly 200 mice that they have experimented with. Importantly, they also found that there were no long-lasting effects of the pulsed ultrasound at 24 hours and 7 days post-treatment. This means that in its current form, transcranial pulsed ultrasound would be effective at rapidly modulating neuron activity but not chronically changing neuronal firing. Perhaps, this could be as a trial for determining who might benefit from chronic stimulation. Imagine performing transcranial pulsed ultrasound before placing the DBS, proving that stimulation of a particular region produces symptomatic relief prior to the patient undergoing surgery. This is just one possibility with this technology complementing existing techniques.

While the authors clearly demonstrated the ability of transcranial pulsed ultrasound to modulate the activity of underlying cortical neurons, it would be of even greater interest to know whether this technique could be used to activate subcortical structures (that is, structures currently targeted in neuromodulation therapies). To explore this, the authors turned their attention to intact mouse hippocampus. By performing extracellular recording in CA1, they demonstrated that pulsed ultrasound resulted in local field potentials followed by non-epileptogenic after-discharges as well as increased spike frequency. Furthermore, pulsed ultrasound stimulated physiological gamma oscillations and sharp wave ripples, indicating normal hippocampal network activation and synchrony. Translation: this may be able to modulate deep brain structures, including the basal ganglia and thalamus, and with 2-mm precision — well, that‘s pretty great!

### Key Points:

1. Transcranial pulsed ultrasound has the advantages of being noninvasive, having high lateral spatial resolution (~2 mm, though the depth resolution needs to be explored further), and being non-destructive. This could change the practice of neuromodulation entirely, or at least complement existing techniques by allowing the surgeon to noninvasively assay which patients would benefit most from chronic electrical stimulation with DBS.
2. While the results presented are impressive, more work is necessary to define the spectrum of potential translational applications. It would be important to know the three-dimensional volume of tissue being stimulated. It would be interesting to know whether high-intensity stimulation blocks neuronal activation.
3. Noninvasive modes of neuromodulation will be key in the future of functional neurosurgery. Overcoming some of the limitations of DBS (though having some of its own), this technology and other more precise noninvasive or less invasive methods of modifying neural activity will define the future of this field.

## DEVELOPING NEW NEURONS FOLLOWING INJURY? IT IS POSSIBLE.[3]

Current neuroscience principles dictate that adult neurogenesis (development of new neurons) is restricted to the dentate gyrus of the hippocampal formation and subventricular zone (the latter being involved in the rostral migratory stream to the olfactory bulb). There have been many reports of adult neurogenesis occurring *normally* in other anatomic regions as well as in response to certain disease states. Many of these studies are deemed controversial and require further investigation before being accepted by the entire neuroscience community. In this study, Vessal *et al*. explore cortical neurogenesis in primates following cervical dorsal rhizotomy (DRL). They have previously demonstrated that neurogenesis occurs in the cervical spinal cord following this injury.

As a result of previous findings demonstrating topographic reorganization of the hand region in the sensorimotor cortex after DRL, the authors examined cellular changes within this region following injury. After functional deafferentiation of the thumb, index and middle fingers, the animals were injected with anterograde tracers into their S1 (sensory) hand cortex. These tracers are incorporated into the sensorimotor cortical neurons and fill the entire cell body, allowing for detailed visualization of the sensory neurons and seeing their projections. Then, the animals were injected twice with BrdU, a thymidine analog that allows for visualization of mitotically active cells by integrating into DNA that undergoes replication during the S phase of cell division. Four weeks later, the primates were sacrificed and their brains examined.

Compared to the ipsilateral (control) hemisphere, the contralateral hand S1 cortex showed a significantly greater number of BrdU-labeled cells. The immature new cells expressed were labeled by BrdU only, while more mature neurons were also labeled by markers for NeuN or calbindin. These mitotically-active cells were most prevalent in the reorganized region of the hand, S1, an area that already was known to undergo changes following injury. Interestingly, while the BrdU labeling was much greater on the contralateral hemisphere, the ipsilateral (control) hemisphere did show minimal BrdU labeling. This may be due to the bilateral representation of the hand in the S1 cortex, signifying that neurons projecting to both sides undergo some new cell division. That being said, no BrdU labeling was present in the cortex of a non-lesioned control monkey.

When comparing the cell types of BrdU-labeled cells, approximately 34% of labeled cells were neurons within the more superficial laminae (2 and 3). These neurons were reconstructed using the anterograde tracer, and both apical and basal dendrites were observed in the nascent pyramidal neurons. Further analysis demonstrated that more than half of the neurons were calbindin-positive, suggesting their identity as interneurons. Interestingly, these calbindin-positive interneurons were not GAD67-positive, implying they were excitatory and not inhibitory (suggesting an immature phenotype). This neurogenesis appears to be restricted to the hand S1 region, as sections from prefrontal and posterior parietal cortices failed to demonstrate BrdU+ cells. So the question is, are these BrdU-labeled cells a result of local neurons that are dividing in response to growth factors released following injury? Or are they migrating from somewhere else (i.e., the subependymal zone)? An even more interesting question is, if sensory cortical neurons retain the capacity to divide in response to certain stimuli, can we harness this ability to reproduce neurons in particular regions following brain injury?

### Key Points:

Adult neurogenesis may occur following neurological injury, specifically in regions that undergo architectural reorganization in response to a lesion. In this case, the S1 cortex showed signs of new neurons following deafferentiation.

BrdU labeling provides a method for assaying mitotically-active cells postmortem by injecting systemically prior to sacrifice.

The extent of adult neurogenesis, both physiologically and in response to injury, as well as the extension of this concept to the human brain, is still uncertain. It does, however, provide some insight into the retained capacity of cells to undergo division when prodded by particular environmental stimuli.
